# Human papillomavirus and human telomerase RNA component gene in cervical cancer progression

**DOI:** 10.1038/s41598-019-52195-5

**Published:** 2019-11-04

**Authors:** Yang Liu, Pianping Fan, Yingying Yang, Changjun Xu, Yajuan Huang, Daizhu Li, Qing Qing, Chunyi Sun, Honglin Zhou

**Affiliations:** 1 Department of Reproductive, the Second Affiliated Hospital of Kunming Medical University, Kunming, Yunnan 650101 P.R. China; 2 Department of Gynecology, LinFen Central Hospital of Shanxi Province, LinFen, Shanxi 041000 P.R. China; 3 Department of Gynecology, the Second Affiliated Hospital of Kunming Medical University, Kunming, Yunnan 650101 P.R. China

**Keywords:** Diseases, Oncology

## Abstract

This study aimed to examine hTERC gene in different grades of cervical intraepithelial neoplasia (CIN) and cervical cancer, and the association between hTERC and high risk-human papillomavirus (HR-HPV) infection. Patients who underwent cervical cancer screening at the Second Affiliated Hospital of Kunming Medical University between October 2010 and December 2011 were enrolled. All patients underwent liquid-based cytology test and hybrid capture 2 (HC2) for HPV detection. hTERC was examined using fluorescence *in situ* hybridization (FISH). Cervical colposcopy biopsy was performed if any of the three results was positive. HC2, FISH, and pathology were compared. A total of 1200 women underwent screening, 150 patients underwent cervical biopsy: 32 in the normal group, 38 in the CIN1 group, 66 in the CIN2/3 group, and 14 in the invasive cervical cancer group. More patients had HR-HPV infection in the CIN2/3 group and ICC group compared with the CIN1 group. hTERC increased with increasing histological dysplasia. There was significant difference in hTERC positive rate between each of the three groups. More patients with hTERC gene amplification were observed in the positive HR-HPV group than in the HR-HPV negative group. In conclusion, hTERC is a potential marker for precancerous cervical cancer lesions. hTERC might be correlated with HR-HPV infection in cervical diseases.

## Introduction

Approximately 525,000 people worldwide are being diagnosed with cervical cancer each year^1,2^, and 274,000 people are dying of cervical cancer, for a mortality rate of 7.8/100,000^3^. Cervical cancer in China accounts for 12% of the worldwide cervical cancer incidence and for 11% of the cervical cancer deaths^4^. In countries that develop and implement cervical cancer screening, the incidence of cervical cancer has been significantly reduced by regular follow-up and monitoring screening quality^5^. But in many areas there are no standardized procedures or conditions to perform cervical cancer screening. In China, screening varies according to region, and barely reaches 19% of eligible rural women, resulting in increasing numbers of patients with cervical cancer^4^.

The occurrence and development of cervical cancer takes about 8–12 years from high-grade precancerous lesions to invasive cervical cancer^6^. The precancerous lesions maintain for a long time and can be reversed, so identification of cervical intraepithelial neoplasia (CIN) together with reasonable and effective early treatment are the keys to reducing the incidence and mortality of cervical cancer^7^. Human papillomavirus (HPV) infection is closely related to cervical cancer^8^. High risk-HPV (HR-HPV) can be detected in cervical cells of 99.8% of patients with cervical cancer^9^. Although the sensitivity of HPV-DNA detection is high, the specificity is not high, especially for young women with an active sexual life. Most HR-HPV infections are transient and disappear after 8–10 months, with only a small percentage of HR-HPV being persistent and eventually developing to cervical cancer^10^. Only long-term or repeated infection of HR-HPV may lead to unlimited proliferation of cells, greatly increasing the incidence of cervical cancer^11^. The low specificity of HPV detection limits its clinical application, and more reliable screening methods are needed.

Telomeres are repetitive nucleotide sequences at the end of the chromosomes that maintain the stability of chromosomes and protect genes. These sequences are added by telomerase which contains a reverse transcriptase consisting of an RNA component, a reverse transcriptase and telomerase-related proteins^12^. The human telomerase RNA component (hTERC) acts as a template during telomere elongation but is also a vital part of the telomerase enzyme function^13^. Mutation, activation, and amplification of the hTERC gene may lead to increased telomerase activity^14^. Telomerase reactivation or upregulation is a critical feature in the vast majority of cancers^15^. Expression of hTERC gene is consistent with telomerase activity. hTERC is expressed in many normal tissues and benign tissues, but its relative up-regulation can reflect the degree of malignant progression and it is highly expressed in malignant tumors^16^. In cervical cancer and precancerous lesions, hTERC also has different degrees of amplification, and it has been considered to be a potential tumor marker for cervical cancer^17^.

The HPV proto-oncoprotein encoded by its oncogene E6/E7 can up-regulate telomerase activity in cervical cancer and cervical intraepithelial neoplasia after HR-HPV infection, so that undifferentiated epithelial cells continue to proliferate^18^. HR-HPV infection can lead to hTERC gene amplification^19^. There is no activation of telomerase in the early stage of cancer induced by HR-HPV infection, but it tends to be consistent with the increasing histological dysplasia. Therefore, it is believed that there are sequential differences in the process of cervical carcinogenesis. So, HR-HPV infection is the initiation factor leading to reactivation of hTERC, and the combined action of the two result in cervical cells gaining unlimited proliferation ability^20^. There is a close correlation between HR-HPV infection and activation of the hTERC gene in the development of cervical cancer^21–23^. Increased HR-HPV and hTERC are related to more aggressive disease and may be important parts of future screening tests^21,22^.

The aim of this study was to explore the role of the hTERC gene as a biomarker in cervical cancer. Therefore, hTERC gene expression and HR-HPV infection in exfoliated cervical cells of normal, CIN and ICC patients were detected, and the relationship between the two was analyzed.

## Results

### HR-HPV infection and hTERC gene amplification

During the study period (October 2010 to December 2011), 1200 women participated in the cervical cancer screening program and 150 (12.5%) underwent cervical biopsy, including 32 (21.3%) in the normal group, 38 (25.3%) in the CIN1 group, 66 (44.0%) in the CIN2/3 group, and 14 (9.3%) in the ICC group. The HR-HPV infection rate was significantly different among the four groups (P < 0.001). There were more patients with HR-HPV infection in the CIN2/3 (90.9%) and ICC groups (92.9%) compared with the CIN1 group (65.8%, P < 0.05) and normal group (31.3%, P < 0.05). There was no difference in HR-HPV infection between the CIN2/3 and ICC groups (P = 0.864) (Table 1).

**Table 1:** HR-HPV positive rate and hTERC positive rate.

Variable	Normal (n = 32)	CIN1 (n = 38)	CIN2/3 (n = 66)	ICC (n = 14)	P
HR-HPV+	10 (31.3)	25 (65.8)	60 (90.9)	13 (92.9)	<0.001
hTERC+	4 (12.5)	13 (34.2)	35 (53.0)	12 (85.7)	<0.001
HR-HPV+ & hTERC+	2 (6.3)	12 (31.6)	34 (51.5)	12 (91.7)	<0.001

HR-HPV = high-risk human papillomavirus, hTERC = human telomerase RNA component, CIN = cervical intraepithelial neoplasia, ICC = invasive cervical carcinoma.

With increasing histological dysplasia, patients with hTERC gene amplification showed a significant increasing trend (P < 0.001, Fisher’s exact test). Patients with normal cervical histology had a rate of 12.5% of hTERC amplification. This rate increased to 34.2% for CIN1, 53.0% for CIN2/3, and 85.7% for ICC. There was significant difference in the hTERC positive rate between the CIN1 and CIN2/3 groups, between the CIN1 and ICC groups, as well as between the CIN2/3 and ICC groups (P < 0.05) (Table 1). Regarding the co-occurrence of HR-HPV and hTERC amplification, the double-positive rate was 6.3% in the normal group, 31.6% for CIN1, 51.5% for CIN2/3, and 91.7% for ICC (P < 0.001) (Table 1).

### Association of hTERC gene amplification with HR-HPV infection

In order to examine the association between hTERC gene expression and HR-HPV infection, the patients were also grouped according to the HR-HPV infection status. Compared with the negative HR-HPV group, more patients with hTERC gene amplification were observed in the positive HR-HPV group (54.6% vs. 11.9%, P < 0.001) (Table 2).

**Table 2:** hTERC gene amplification in patients with positive or negative HR-HPV.

Variable	HR-HPV + (n = 108)	HR-HPV− (n = 42)	P
hTERC+	59 (54.6)	5 (11.9)	<0.001

HR-HPV = high-risk human papillomavirus, hTERC = human telomerase RNA component

## Discussion

### Principal findings

This study investigated hTERC in different grades of CIN and cervical cancer, and the association between hTERC and HPV infection. The results showed that hTERC increased with increasing histological dysplasia and there was a significant difference in the hTERC positive rate between each of the three groups. 90.9% patients had HR-HPV infection in the CIN2/3 group, 92.9% in the ICC group and 65.8% in the CIN1 group. More patients with hTERC gene amplification were observed in the positive HR-HPV group than in the HR-HPV negative group. These results suggest that hTERC is a potential marker for precancerous cervical cancer lesions and that hTERC might be correlated with HR-HPV infection in cervical diseases. Nevertheless, some patients had lesions that were negative for both markers. This highlights that even if HR-HPV+ and hTERC amplification are involved in the pathogenesis of ICC, they are not the only factors involved and additional studies are necessary to identify what is driving the increasing histological dysplasia in those patients.

### Results of the study in the context of other research

HPV somatotype is performed based on homology of nucleotide sequence^24^. More than 100 genetic types have been identified, including more than 40 types that can invade the female reproductive tract. These are divided into high-risk and low-risk types based on their carcinogenicity. Many laboratory studies and epidemiological investigations have shown that the main cause of cervical cancer is HR-HPV infection, and there is a chronological sequence for HR-HPV infection and cervical cancer^25^. The most sensitive method for diagnosing cervical lesions is HPV-DNA. Some studies have shown that 95% of patients with cervical cancer are infected with HPV^26^. PCR was used to detect HPV in 1918 cervical cancer specimens from nine countries, and the results demonstrated that the detection rate of HPV is as high as 96.6%^27^. The results of this study showed that HR-HPV infection rates in the normal, CIN1, CIN2/3, and ICC groups were 31.3%, 65.8%, 90.9%, and 92.9%, respectively. The differences among the groups were statistically significant (except between the CIN2/3 and ICC groups). The results showed that the HR-HPV infection rate increased with the increasing histological dysplasia of the cervical lesions, which is consistent with previous results^21^. Moreover, the results demonstrated that HR-HPV infection was not statistically significant between CIN2/3 and ICC groups.

The chromosomes of most solid tumor cells in humans often exhibit certain instability such as changes in the number of chromosomes and structural abnormalities^28^. Most studies have shown that the development of cervical cells from CIN1, CIN2, and CIN3 to cervical cancer is basically accompanied by the amplification of the long arm of chromosome 3^29^. The gene most closely related to cervical cancer may be the hTERC gene. hTERC gene amplification can prevent cell apoptosis, leading to unlimited cell proliferation. Therefore, abnormal amplification of the hTERC gene may be an early event in tumor formation^12^. Heselmeyer-Haddad *et al.*^30^ used FISH trichrome probes in cells with cervical lesions; the 3q26 hTERC gene was amplified at different degrees in CIN2, CIN3, and cervical cancer. Moreover, normal cells, ASC-US, CIN1, and CIN2/3 were distinguished according to the degree of amplification of the hTERC gene. Tu *et al.*^31^ revealed that the amplification rate (4.6%, 13.5%, 28.6%, 25.2%, and 87.7%) of hTERC increases gradually in negative for intraepithelial lesions or malignancy (NILM), ASC-US, atypical squamous cell-cannot exclude high grade intraepithelial lesion (ASC-H), low grade squamous intraepithelial lesion (LSIL), and high grade squamous intraepithelial lesions (HSIL). Except for ASC-H and LSIL, pair-wise comparison was statistically significant. From these two studies, Tu *et al.*^31^ have concluded that the hTERC gene is a better method for differentiating between high- and low-grade lesions both cytologically and histologically. The results of this study showed that the positive rates of the hTERC gene in the normal, CIN1, CIN2/3, and ICC groups were 12.5%, 31.21%, 53.03%, and 85.71%, respectively. Compared to the normal group, there were significant differences in the CIN1, CIN2/3, and ICC groups (*P* < 0.05). There were significant differences between the CIN1 and CIN2/3 groups, between the CIN1 and ICC groups, and between the CIN2/3 and ICC groups. With the increasing histological dysplasia, the positive expression of hTERC gene showed a significantly increasing trend. The results were consistent with Tu *et al.*^31^ and later studies that also suggested that hTERC gene detection can detect cervical cancer in an early stage and may predict lesion progression^21–23^.

HPV infection is closely related to the activation of telomerase. Studies on the carcinogenic mechanism of HPV show that in cervical cancer and cervical intraepithelial neoplasia, the proto-oncoprotein encoded by the oncogene E6/E7 can up-regulate telomerase activity after HR-HPV infection, leading to continuous proliferation of epithelial cells. Ohta *et al.*^32^ used HR-HPV 16 to transfect normal human cervical epithelial cells. These transfected cells were passaged for more than 100 generations and had strong telomerase expression, suggesting that HPV infection is correlated with activation of telomerase. HR-HPV infection may lead to amplification of the hTERC gene^33^. HR-HPV infection is not accompanied by the activation of telomerase in the early stage of cervical cancer, but with the increasing histological dysplasia, the two become more consistent. There is a sequence in the process of cervical carcinogenesis, which means that HR-HPV infection causes the re-activation of the telomerase gene, resulting in unlimited proliferation of cervical cells^12^. Virus oncogene protein E6 of HPV may have a certain correlation with telomerase activation, and up-regulation of hTERC gene expression during telomerase activation may be the main pathway.

In this study, the HR-HPV rates in the normal, CIN1, CIN2/3, and ICC groups were 31.25%, 65.79%, 90.91%, and 92.86%, respectively. With increasing histological dysplasia of the cervical lesions, HR-HPV infection was aggravated, and hTERC gene expression was also increased, thus the two tended to be consistent. In this study the positive rate of hTERC was 54.63% (59/108), while the negative rate was 45.37% (49/108) in the HPV positive group. Whereas, the positive rate of hTERC was 11.90% (5/42) and the negative rate was 88.10% (37/42) in the HPV negative group. The difference was statistically significant, suggesting that that there was a correlation between hTERC gene amplification and HR-HPV infection, which is consistent with previous studies^21,22^.

### Clinical implications

These results add to the growing evidence for the usefulness of hTERC as a biomarker in cervical cancer and precancerous lesions. But, because only long-term or repeated infection of HR-HPV may lead to hTERC gene amplification and the infinite proliferation of cervical cells, there are patients in this study without hTERC amplification in the HR-HPV infection group. hTERC is expressed in many normal and benign tissues, but up-regulation can reflect the degree of malignant progression of tissues and it is highly expressed in malignant tumors. Telomerase activity is closely related to the development of tumors. The hTERC gene plays an important role in telomerase activity. Therefore, inhibiting telomerase activity through hTERC might be used as a new method for treating tumors.

### Research implications

The activation and regulation of a proto-oncogene is a very complex and continuous process. The occurrence and development of cervical lesions can be induced and activated by one gene, so the study of hTERC and its relationship with HPV infection needs to be further verified in more detailed studies.

### Strengths and limitations

The strength of the study lies in the 1200 women who underwent screening and the inclusion of different grades of CIN and cervical cancer. The study is limited because it was a single center and the groups were relatively small. In addition, the potential role of HPV types in hTERC positivity was not explored. Larger studies from multiple centers are needed to fully evaluate the role of hTERC as a biomarker for cervical cancer.

## Conclusions

Significant hTERC gene amplification rates were found in precancerous cervical lesions and these rates increased with the increasing histological dysplasia of the cervical lesions through to ICC. This suggests that hTERC can be used as a biomarker for precancerous lesions of cervical cancer and might be expected to become an early screening method for cervical cancer. The cervical lesions were also associated with HR-HPV infection and there was a certain correlation between hTERC gene and HR-HPV infection.

### Subjects and methods

#### Patients

Outpatients and inpatients who voluntarily participated in cervical cancer screening at the Department of Gynecology of the Second Affiliated Hospital of Kunming Medical University between October 2010 and December 2011 were enrolled. All women had a history of sexual activity; were not pregnant; had not undergone colposcopy biopsy before; and were cervical treatment-naive. Each woman underwent a liquid-based cytology test (LCT), hybrid capture 2 (HC2)-HPV test and hTERC gene test. Cervical colposcopy biopsy was performed if LCT ≥ atypical squamous cell of undetermined significance (ASC-US) or positive HPV (≥1.0 pg/ml) or hTERC gene ≥ threshold determined from women with normal samples as described later. Two pathologists participated in the assessment. This study was approved by the Ethics Committee of the Second Affiliated Hospital of Kunming Medical University. Informed consent was waived due to the nature of retrospective study.

#### Collection of LCT specimens

A brush for LCT cell collection was inserted into the junction of the squamous epithelium and columnar epithelium of the external cervical orifice. With the external cervical orifice as the center, the brush was removed after completely rotating evenly 5–7 times. Then, the brush head was placed in the LCT cell preservation solution and shaken well, letting the collected cells disperse into the preservation solution and sent to the pathology department for detection. Cells remaining in the preservation solution were investigated for hTERC gene expression.

#### Collection of HPV-DNA specimens

A cervical sampling brush for HC2 was inserted into the cervical canal, which was rotated in the same direction 3–5 time to collect exfoliated cervical cells. Then, the specimens along with the brush were placed in the preservation solution to be sent for gynecological examination at our hospital using the HC2 HR-HPV Test Kit (Digene Corporation, Gaithersburg, MD, USA).

#### hTERC gene detection using fluorescence *in situ* hybridization

Cervical cells were isolated from 5–10 ml of LCT cell preservation solution and treated with 3 ml of collagenase B solution at 37 °C for 20–30 min. The cells were pelleted and mixed with 5 ml of deionized water preheated to 37 °C and incubated at 37 °C for 20 min with occasional mixing. Stationary liquid (2 ml) was slowly mixed into the hypotonic mixture. The pellet was harvested and washed twice with 5 ml of stationary liquid for 10 min. The final pellet was resuspended in fixative solution and spread on a glass slide. After the slides had dried they were washed twice with 2× saline sodium citrate (SSC) solution (5 min each), then soaked in 0.1 mol/L hydrochloric acid solution for 10 min, 0.02 mg/ml pepsin working solution at 37 °C for 10 min, and then washed twice with 2× SSC solution (pH 7.0) (5 min each). The samples were dehydrated sequentially in 70% ethanol, 85% ethanol, and 100% ethanol, dried and then heated to 56 °C. The GLP hTERC/chromosome 3 centromere (CSP3) DNA probe mixture (10 μl) (Beijing Jinpujia Medical Technology Co., Ltd.) was added, then immediately covered with a coverslip and sealed with rubber. Co-denaturation was performed at 75 °C for 8 min, then hybridization at 42 °C for 16 h. The coverslips were removed, and the glass slides were placed in 0.3% NP-40/0.4× SSC solution at 67 °C with shaking for 1–3 s, then rinsed for 2 min. The slides were then in 0.1% NP-40/2× SSC solution and shaken for 1–3 s, and rinsed for 30 s. The slides were placed in 70% ethanol, and rinsed for 3 min. DAPI re-staining agent (15 μl) was added to the target area, then covered with coverslips and kept in the dark for 10–20 min. Before observation with light microscopy. The whole hybridization area was scanned under a 40× objective lens and the quality of the specimens was observed. Satisfactory specimens had over 75% of the nuclei with hybridization signals. hTERC gene amplification in cervical cells was then observed under a 100× objective lens, and the signals were counted. The GLP hTERC specific probe was labeled with red fluorescence. The CSP3 probe labeled with green fluorescence was used as the control.

**Figure 1:**
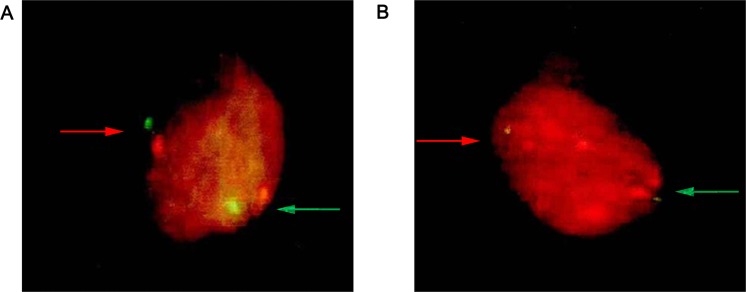
Expression of hTERC gene in cervical cells under fluorescence microscope (100X). (**A**) Normal cell; (**B**) Cell with hTERC gene amplification. Red signal: hTERC; Green signal: CSP3.

Normal cells were those that gave two red and two green signals in a single interphase nucleus (recorded as 2–2, Fig. 1A). Cells with hTERC gene amplification had at least two green signals in a single interphase nucleus, and more than two red signals. Two green dots and four red dots represented that hTERC gene had quadriploidy amplification (recorded at 2–4, Fig. 1B).

The normal threshold was established by samples of cervical cells collected from 20 women with normal pathological examination. Those 20 women were randomly selected in the process of a quality control process from the population of women who were tested at our center in 2018. One hundred cells from each sample were analyzed to calculate the percentage of cells with hTERC gene amplification. According to the calculation: Threshold = mean + 3 × standard deviation (SD), the hTERC gene threshold in this study was 6.2%. Then, 100 cells from each study participant were randomly selected. If the percentage of cells was greater than the threshold, then the sample was positive. If the percentage of cells was less than the threshold, then the sample was negative. If the percentage of abnormal cells was equal to the threshold, then another 100 cells were randomly selected for detection.

### Statistical analysis

Statistical analysis was performed using SPSS 17.0 (IBM, Armonk, NY, USA). Categorical variables are expressed as frequency (percentage) and analyzed using chi-square test. P < 0.05 was considered statistically significant.

## Data Availability

The datasets generated during and/or analyzed during the current study are available from the corresponding author on reasonable request.
